# An *in-planta* comparative study of *Plasmopara viticola* proteome reveals different infection strategies towards susceptible and *Rpv3*-mediated resistance hosts

**DOI:** 10.1038/s41598-022-25164-8

**Published:** 2022-12-01

**Authors:** Joana Figueiredo, Rita B. Santos, Leonor Guerra-Guimarães, Céline C. Leclercq, Jenny Renaut, Rui Malhó, Andreia Figueiredo

**Affiliations:** 1 Grapevine Pathogen Systems Lab, Plant Biology Department, BioISI – Biosystems & Integrative Sciences Institute, Faculty of Sciences, University of Lisbon, 1749-016 Lisboa, Portugal; 2 Plant Biology Department, BioISI – Biosystems & Integrative Sciences Institute, Faculty of Sciences, University of Lisbon, 1749-016 Lisboa, Portugal; 3 CIFC - Centro de Investigação das Ferrugens Do Cafeeiro, Instituto Superior de Agronomia, Universidade de Lisboa, 1349-017 Lisboa, Portugal; 4 LEAF - Linking Landscape, Environment, Agriculture and Food & Associated Laboratory TERRA, Instituto Superior de Agronomia, Universidade de Lisboa, 1349-017 Lisboa, Portugal; 5 Environmental Research and Innovation Department, Luxembourg Institute of Science and Technology, 4362 Esch-Sur-Alzette, Luxembourg

**Keywords:** Molecular biology, Plant sciences

## Abstract

*Plasmopara viticola*, an obligate biotrophic oomycete, is the causal agent of one of the most harmful grapevine diseases, downy mildew. Within this pathosystem, much information is gathered on the host, as characterization of pathogenicity and infection strategy of a biotrophic pathogen is quite challenging. Molecular insights into *P. viticola* development and pathogenicity are just beginning to be uncovered, mainly by transcriptomic studies. *Plasmopara viticola* proteome and secretome were only predicted based on transcriptome data. In this study, we have identified the *in-planta* proteome of *P. viticola* during infection of a susceptible (‘Trincadeira’) and a *Rpv3*-mediated resistance (‘Regent’) grapevine cultivar. Four hundred and twenty *P. viticola* proteins were identified on a label-free mass spectrometry-based approach of the apoplastic fluid of grapevine leaves. Overall, our study suggests that, in the compatible interaction, *P. viticola* manipulates salicylic-acid pathway and isoprenoid biosynthesis to enhance plant colonization. Furthermore, during the incompatible interaction, development-associated proteins increased while oxidoreductases protect *P. viticola* from ROS-associated plant defence mechanism. Up to our knowledge this is the first *in-planta* proteome characterization of this biotrophic pathogen, thus this study will open new insights into our understanding of this pathogen colonization strategy of both susceptible and *Rpv3*-mediated resistance grapevine genotypes.

## Introduction

The outcome of plant-pathogen interactions depends on mutual recognition, on the activation of pathogenicity and virulence factors by the pathogen, and on the existence of constitutive or inducible defence mechanisms from the host. Interaction may then be classified as compatible (successful infection leading to disease) or incompatible (successful plant defence)^1^. To colonize the host, pathogens secrete proteins, namely hydrolytic enzymes such as proteases, lipases, glycosyl hydrolases, cell wall degrading proteins and inhibitory proteins. The secreted proteins and the cell machinery involved in their secretion are defined as secretome^2^. The main purpose of these secreted proteins is to enable the invasion and colonization of the plant cells and lastly, the digestion of complex substrates into small units that act as nutritional sources^3^. Within the secreted proteins some are described as effector proteins that can either be found at the apoplast when they act at the host’s extracellular environment (i.e. hydrolytic enzymes) or be found at the cytoplasm when they are translocated into host cells (RxLR and Crinkler effectors)^4,5^. Pathogen effectors contribute to modulate metabolic, physiological, and morphological processes in the host plants, thereby facilitating infection and colonization (virulence factors or toxins) and/or triggering defence responses (avirulence factors or elicitors)^6^. In the case of RxLR and Crinkler effectors, they have been reported to induce plant cell death and some of the RxLR effectors are avirulence proteins that exhibit gene-for-gene interactions with specific host resistance proteins and trigger hypersensitive responses (HR) in plants^7,8^.

Within plant pathogens, oomycetes are the etiological agents of many of the world’s most serious plant diseases, being able to breach the intact surfaces of host plants, rapidly establish infection which can result in significant yield losses in large-scale agricultural and horticultural industries^3^. The obligate biotrophic oomycete *Plasmopara viticola* (Berk. et Curt.) Berl. & de Toni is the causal agent of downy mildew, one of the most destructive grapevine (*Vitis vinifera* L.) diseases. The development of highly tolerant grapevine crop varieties through breeding programs is one of the most effective and environmental-friendly disease control strategies to reduce the excessive application of pesticides. However, several studies have shown that, in Europe, some *P. viticola* isolates were able to overcome host resistance mediated by different resistance to *P. viticola* (RPV) loci^9–11^. A comprehensive knowledge of the evolution of pathogen infection mechanisms is essential to improve plant resistance. Moreover, knowing in detail the differences in the infection process during the establishment of compatible and incompatible interactions could help to define new targets for the improvement of the present strategies for disease control.

In recent years, the study of plant pathogens has been greatly promoted by the availability of their genome sequences. The genome of *P. viticola* was already sequenced^12,13^, however, until now only transcriptomic studies have been published and the *P. viticola* proteome and secretome were only predicted. In the present study, a label-free mass spectrometry-based approach was used to tackle the challenge of characterising, for the first time, the *in-planta P. viticola* proteome. Host extracellular compartment was chosen as it is where the pathogen develops most of the infection structures, thus grapevine leaves apoplastic fluid from two grapevine cultivars (‘Trincadeira’—compatible interaction and ‘Regent’—incompatible interaction) infected with *P. viticola* was isolated and *P. viticola* proteome was sequenced at several time-points after infection. Our study highlights the specificity of *P. viticola* proteome regarding host tolerance. Up to our knowledge this is the first report of *P. viticola in-planta* proteome.

## Results

### *Plasmopara viticola* infection assessment

Both phenotypic and two RxLR effectors (*PvRxLR28* and *PvRxLR67*) mRNA levels were assessed to confirm the success of the inoculation assay (Supplementary Fig. S1). The selected RxLR effectors were previously shown to be activated at early (*PvRxLR28*) and late (*PvRxLR67*) inoculation times^14^ in both interactions.

In the compatible interaction between ‘Trincadeira’ and *P. viticola*, the *PvRxLR28* presented a high amount of target mRNA up to 24 h post inoculation (hpi) and then a decrease until 72 hpi. In contrast, for *PvRxLR67*, an increase on the amount of target mRNA during the time course of infection was observed, stabilizing between 24 and 48 hpi. In the incompatible interaction of ‘Regent’-*P. viticola*, a slight decrease in *PvRxLR28* mRNA during the infection development was observed, while for the *PvRxLR67* an opposite profile was observed. Moreover, in this interaction, no amplification of both RxLR effectors mRNA was detected at 120 hpi. In both interactions, no amplification of *PvRxLR28* and *PvRxLR67* mRNA was detected in control samples. The results are in accordance with previous studies where *PvRxLR28* showed to be expressed early on the course of *P. viticola*-grapevine interaction while *PvRxLR67* presented a later expression in this pathosystem^15^. The observed profiles for *PvRxLR28* and *PvRxLR67* corroborated the inoculation success (Supplementary Fig. S1).

### Characterization of *Plasmopara viticola* proteome during host infection

A total of 420 *P. viticola* different proteins were identified in grapevine apoplast, considering all time-points and the two interactions. Of those, 218 were identified in the compatible interaction, 126 being specific to this interaction. In the incompatible interaction, 294 proteins were identified, 202 being specific. Ninety-two proteins were common to both interactions. The highest number of proteins was identified in the incompatible interaction, with exception of 6 hpi (Table 1).

**Table 1:** Number of total proteins (“Total”), secreted proteins (“Secretome”) and effector proteins (“Effectome”) identified at each time-point in: (A) total *P. viticola* proteome; (B) ‘Trincadeira-*P. viticola* interaction (specific and common proteins were considered); and (C) ‘Regent’-*P. viticola* (specific and common proteins were considered).

	Time-points				
	6 hpi	24 hpi	48 hpi	72 hpi	120 hpi
**(A) Total *P. viticola* proteome**					
Total	135	133	181	102	128
Secretome	26	22	34	13	24
Effectome	56	44	74	40	59
**(B) ‘Trincadeira’-*P. viticola* interaction**					
Total	81	37	80	39	64
Secretome	16	9	16	7	16
Effectome	29	15	30	13	28
**(C) ‘Regent’-*P. viticola* interaction**					
Total	60	99	122	69	75
Secretome	12	13	19	7	9
Effectome	32	30	51	29	35

Secretome and effectome prediction tools (SignalP, EffectorP 3.0 predictor and EffHunter Pipeline) were applied to the 420 identified proteins. Seventy-nine proteins presented signal peptide prediction for secretion (19% of the total proteome). One hundred and sixty-four proteins were classified as effectors by both EffectorP and EffHunter Pipeline. Combining both secretome and effectome analysis, a total of 48 proteins were identified as effectors that are secreted, corresponding to 11% of the total identified proteome (Table 1).

**Figure 1:**
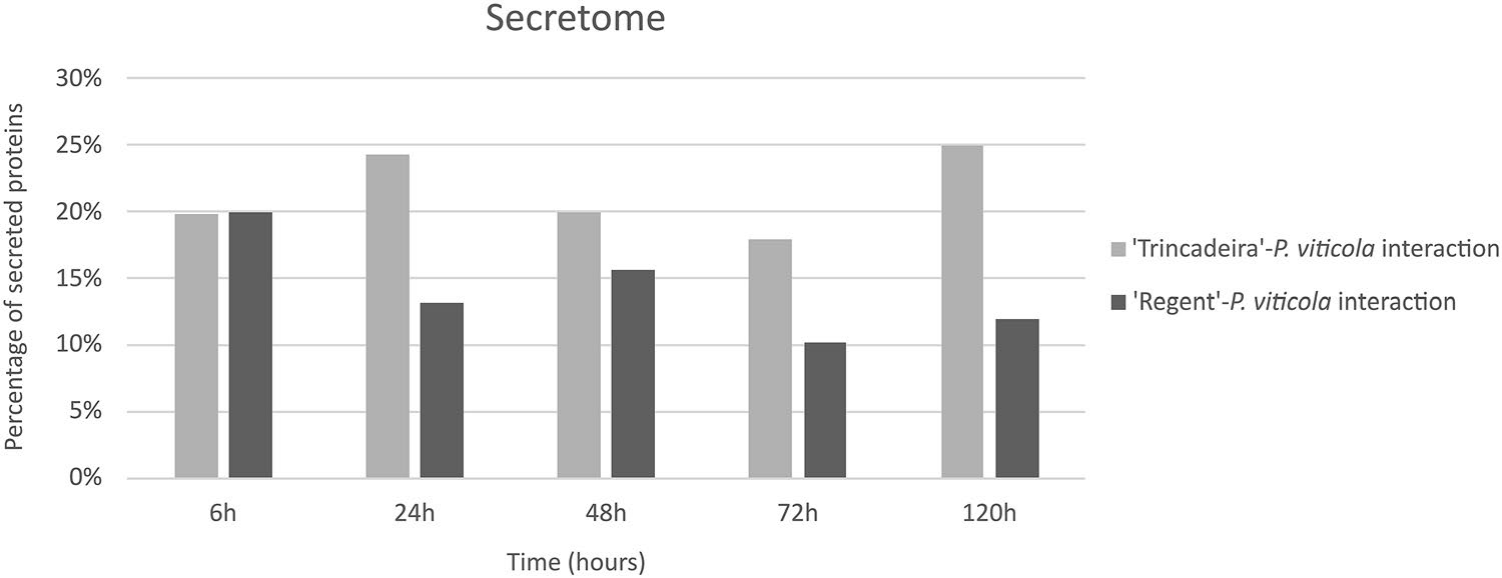
Percentage of *P. viticola* proteins predicted to be secreted during interaction with *V. vinifera* cvs. ‘Trincadeira’ (light grey) and ‘Regent’ (dark grey).

When comparing the percentage of secreted proteins at each time-point, a distinct pattern between interactions is seen. In the compatible interaction, the percentage of secreted proteins was always higher, ranging from 18 to 24%, while in the incompatible was from 10 to 15%, except for 6 hpi, where the percentage of secreted proteins was similar in both interactions (Fig. 1).

**Figure 2:**
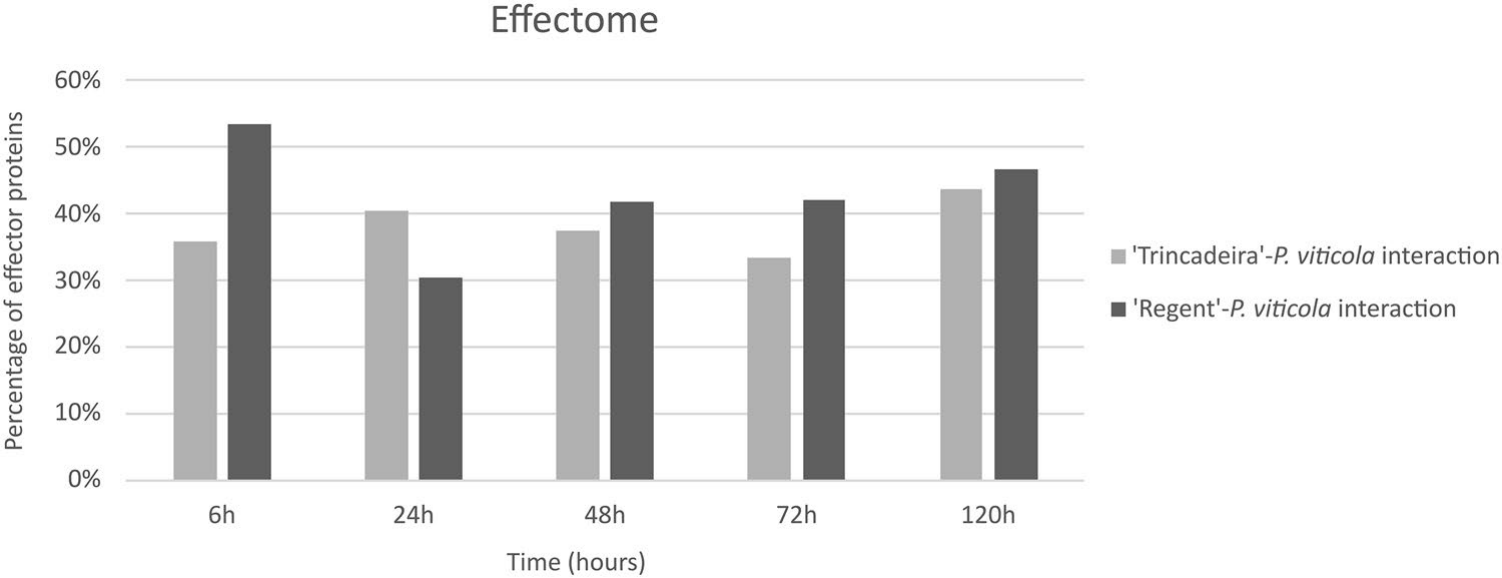
Percentage of *P. viticola* proteins predicted to be effector proteins during interaction with *V. vinifera* cvs. ‘Trincadeira’ (light grey) and ‘Regent’ (dark grey).

The distribution of effector proteins during the time course of infection was also different when *P. viticola* infects susceptible and *Rpv3*-mediated resistance genotypes. In the compatible interaction, the percentage of effector proteins ranged from 33 to 44%, with the maximum value observed at 120 hpi. In the incompatible interaction, the values ranged from 30 to 53%, being the highest percentage observed at 6 hpi. Indeed, at this time-point it was also observed the major difference between both interactions (53% in the incompatible interaction and 36% in the compatible interaction), (Fig. 2).

### Identification of *Plasmopara viticola* protein domains

**Figure 3:**
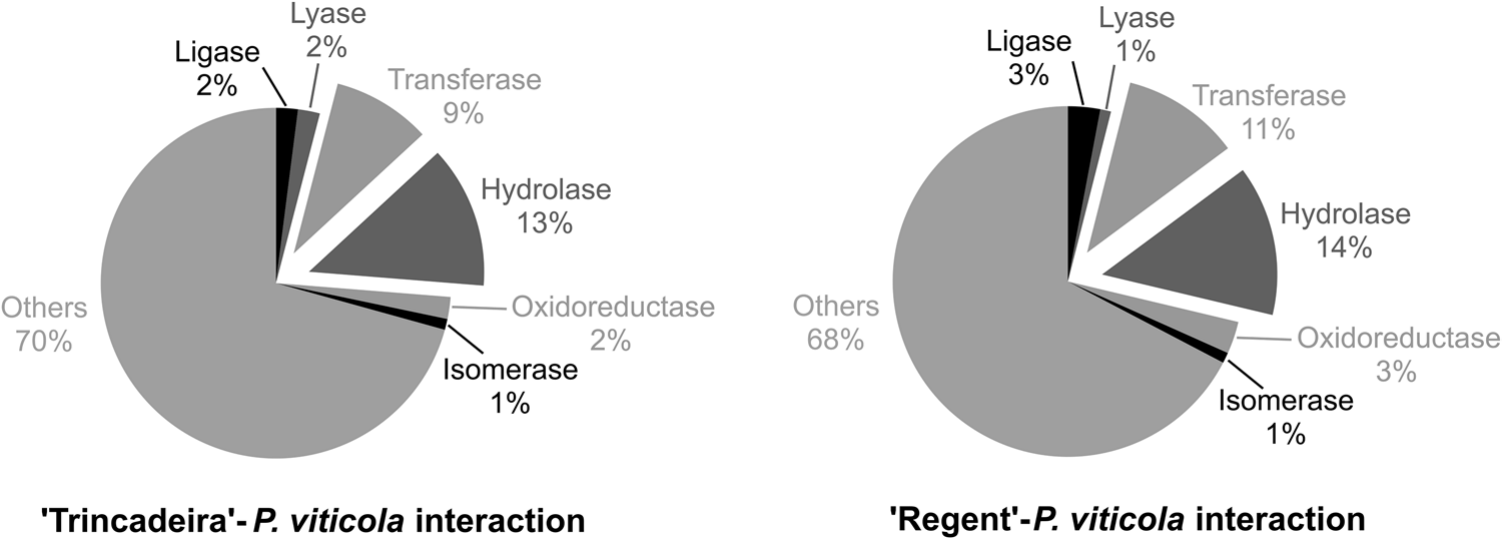
Percentage of *P. viticola* proteins categorized in the seven enzymatic classes.

To further characterize *P. viticola* proteins, a domain search was conducted on the *P. viticola* proteome. Seven classes were identified: (1) hydrolases (subclasses: peptidases and other hydrolases), (2) transferases (subclasses: kinases and other transferases), (3) oxidoreductases, (4) isomerase, (5) ligases, (6) lyases and (7) other proteins (not enzymes or proteins without domain prediction), (Supplementary Table S1). Both interactions presented similar percentages of each class (Fig. 3), being the classes (1) and (2) the most predominant. Within the hydrolase class, around 20% of the proteins were identified as peptidases and, in the transferases class, the majority (50–55%) were identified as kinases based on the presence of a kinase domain.

**Figure 4:**
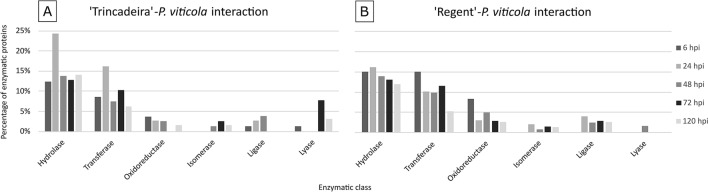
Percentage of *P. viticola* proteins, categorized in the seven enzymatic classes and distributed during the time-course of infection in the compatible (**A**) and in the incompatible (**B**) interactions.

The distribution of the proteins, based on their enzyme classification, along the time course of the infection, was evaluated for both interactions. In ‘Trincadeira’-*P. viticola* interaction, the most representative classes were (1) and (2) (hydrolase and transferase), in all the time points (Fig. 4A). No oxidoreductases were identified at 72 hpi and lyases were only identified at 6, 72 and 120 hpi. Isomerases were only detected at the later time-points while ligases were identified at early time-points, being the 48 hpi the only time-point where both classes were identified (Fig. 4A). In ‘Regent’-*P. viticola* interaction, a higher percentage of hydrolases and transferases was also observed at all time-points, comparatively to the other enzyme classes (Fig. 4B). Oxidoreductases were identified also in all the time-points with the highlight for the 6 hpi. In contrast, isomerases and ligases were not detected at 6 hpi and lyases were only observed at 48 hpi (Fig. 4B).

To identify the type of effector proteins in *P. viticola* proteome, a search for specific motifs was performed. For the prediction of the RxLR-EER motif, present in RxLR effectors, and the LFLAK-HVLV motif, for Crinkler effectors, the *effectR* package was used^16^. Two RxLR effectors (PVIT_0014142.T1 and PVIT_0015177.T1) were found within the effectors present in the incompatible interaction. Four Crinkler effectors were found: PVIT_0001451.T1 and PVIT_0006424.T1 in the interaction with ‘Trincadeira’; PVIT_0006190.T1 in the interaction with ‘Regent’; and PVIT_0025443.T1 was found in both. These proteins were also confirmed to be effectors by the presence of the Crinkler effector protein N-terminal domain (PF20147), (Supplementary Table S1). However, one of these Crinkler effectors presents a modification in one of the amino acids from the conserved motif. Instead of the usual phenylalanine (F) in the second position of the motif, PVIT_0006424.T1 has a tyrosine (Y). Other conserved motifs found in effector proteins like, RGD, RSIDELD, CHXC, RYWT^17^ and RWPxRK^18^ motifs were predicted. In *P. viticola* proteome during interaction with ‘Trincadeira’ and ‘Regent’ none of these effector motifs was predicted.

### *Plasmopara viticola* proteins common to compatible and incompatible interactions

Ninety-two proteins were found to be common to both interactions (Supplementary Table S1). These were divided into three major groups: (1) proteins that were detected in only one time-point, common to both interactions (13 proteins); (2) proteins that were detected in different time-points on each interaction (48 proteins); (3) proteins that were detected in several time-points but were observed in both interactions at least in one of the time-points (31 proteins).

Considering group 1, proteins that were detected at the same time-point in both interactions, a difference in abundance between interactions were observed (Supplementary Table S1). At 48 hpi, an acetyl-CoA carboxylase (PVIT_0002749.T1) showed a higher abundance in ‘Trincadeira’-*P. viticola* interaction (Supplementary Table S2). In contrast, other proteins showed a higher abundance in ‘Regent’-*P. viticola* interaction, such as, a member of the short-chain dehydrogenases/reductases family (PVIT_0010364.T1), a STAG domain-containing protein (PVIT_0007744.T1), a fibroblast growth factor receptor 1 (FGFR1) oncogene partner (FOP) protein (PVIT_0001847.T1) and a homologous to the E6-AP carboxyl terminus (HECT) domain containing protein (PVIT_0000553.T1), (Supplementary Table S2). Only four proteins with effector prediction were identified in group 1 (PVIT_0007295.T1; PVIT_0010364.T1; PVIT_0010515.T1; PVIT_0013696.T1), one of them the previously referred member of the short-chain dehydrogenases/reductases family (Supplementary Table S1).

Within proteins from group 2, most proteins were observed in early time-points in the compatible interaction of *P. viticola* with ‘Trincadeira’ while in the incompatible interaction they were detected at later time-points (Supplementary Table S3). As an example, a tRNA synthetase (PVIT_0006618.T1), a serine/threonine/tyrosine protein kinase (PVIT_0001990.T1) and a phosphate transport (Pho88) domain-containing protein (PVIT_0005502.T1) were identified with higher abundance as soon as 6 hpi in the compatible interaction. In the incompatible interaction these proteins were only detected at 48 hpi (Supplementary Table S3). In contrast, a member of the sodium/hydrogen exchanger family (PVIT_0005313.T1) and an ankyrin domain-containing protein (PVIT_0008845.T1) were also identified at 6 hpi in ‘Trincadeira’-*P. viticola* interaction and at 48 hpi in ‘Regent’-*P. viticola* interaction, but with higher abundance in the latter (Supplementary Table S3). Also, a DNA polymerase (PVIT_0004400.T1) and an elongation factor Tu (PVIT_0011238.T1) were highly abundant in the ‘Trincadeira’-*P. viticola* interaction at 6 hpi, while in the incompatible interaction these proteins were only detected at 72 and 120 hpi, respectively, with a low abundance (Supplementary Table S3).

Also, within group 2, several proteins were detected at early time points in the ‘Regent’-*P. viticola* incompatible interaction, while in the compatible interaction they were detected at later time-points (Supplementary Table S4). This pattern was observed for the serine/threonine/tyrosine protein kinase (PVIT_0013015.T1) and an ubiquitin carboxyl-terminal hydrolase (PVIT_0001556.T1), (Supplementary Table S4). A myotubularin-like phosphatase domain-containing protein (PVIT_0003475.T1) and the Gtr1/RagA G protein of the Ras family (PVIT_0005567.T1) were detected in the incompatible interaction at 24 hpi, while in the compatible interaction they were only detected at 48 hpi (Supplementary Table S4). Moreover, a member of the beta-glucan synthesis-associated proteins SKN1/KRE6/Sbg1 family (PVIT_0017117.T1) and a mitochondrial carrier protein (PVIT_0007479.T1), were identified in ‘Regent’-*P. viticola* interaction at 48 and 72 hpi. In contrast, in the compatible ‘Trincadeira’-*P. viticola* interaction, they were only detected at 120 hpi. The first one showed a higher abundance in the incompatible interaction while the peak of abundance of the mitochondrial carrier protein was detected in the compatible interaction (Supplementary Table S4).

Considering group 3, a member of the nucleoporin Nup120/160 family (PVIT_0016228.T1) was detected in all time-points during the ‘Regent’-*P. viticola* interaction. However, in the compatible interaction, this protein was only detected at 24 and 48 hpi (Supplementary Table S1). A member of the structural maintenance of chromosomes (SMC) superfamily (PVIT_0010749.T1) was detected in all time-points in the incompatible interaction, with exception of 48 hpi. However, in the compatible interaction, this protein was only observed at later time-points as 72 and 120 hpi (Supplementary Table S1). Moreover, two kinases (PVIT_0006222.T1; PVIT_0010368.T1) were detected at 24 and 48 hpi in ‘Regent’-*P. viticola* interaction, while in the other interaction they were detected at 48 and 72 hpi (Supplementary Table S1). Furthermore, a Crinkler domain-containing protein (PVIT_0025443.T1) was observed at later time-points (72 and 120 hpi) and with low abundance in the ‘Regent’-*P. viticola* interaction. In the ‘Trincadeira’-*P. viticola* interaction, this protein appeared also at 24 hpi (Supplementary Table S1).

### *Plasmopara viticola* proteins specifically found in the compatible interaction with ‘Trincadeira’

One hundred and twenty-six proteins were exclusively present in the interaction between *P. viticola* and the susceptible grapevine genotype ‘Trincadeira’. Twenty-nine proteins were predicted to be secreted and, of those, 14 were predicted to be effector proteins. Proteins classified as hydrolases and transferases (excluding peptidases and kinases, respectively) were detected in all the time-points (Supplementary Table S1).

**Figure 5:**
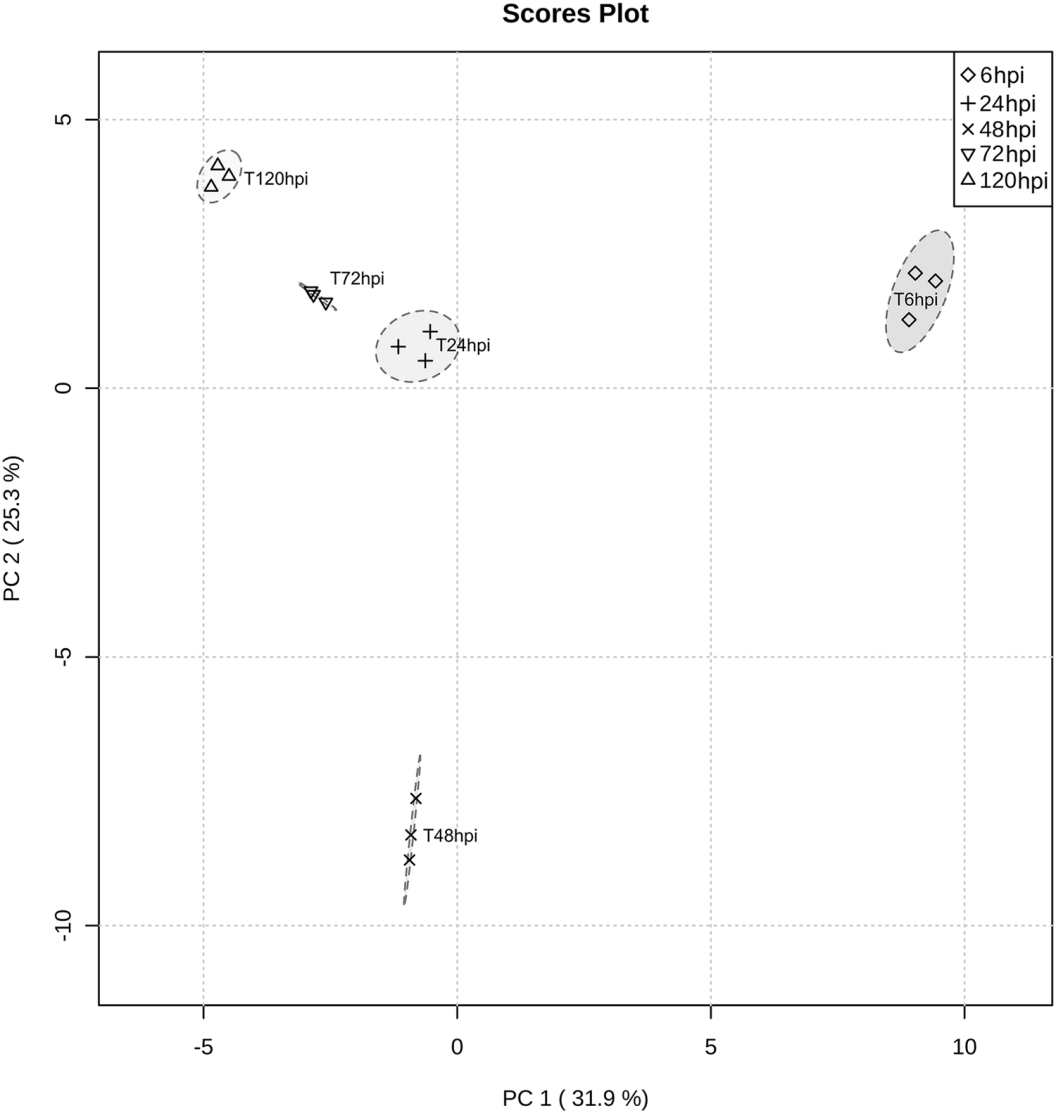
Principal components analysis (PCA) PC1/PC2 score plot of specific *P. viticola* proteome during interaction with *V. vinifera* cv. ‘Trincadeira’. The three biological replicates per condition are represented.

When considering the proteins specific to this interaction, the unsupervised PCA bi-plot revealed that 6 and 48 hpi were the most distinct time-points, showing the highest levels of variation (56%), while the other time-points cluster together (Fig. 5). Thirty-eight proteins are contributing to the distinction of the proteome identified at 6 hpi, and 20 proteins for the 48 hpi proteome. Within these proteins, a Syja domain-containing protein (PVIT_0014411.T1), protein involved in the pathogen phosphoinositide pathway, a polyprenyl synthetase (PVIT_0015689.T1), enzyme responsible for synthesis of isoprenoid compounds, an ENHANCED DISEASE RESISTANCE 2 (EDR2) domain containing protein (PVIT_0008910.T1) and NUDIX domain containing protein (PVIT_0025277.T1), negative regulators of plant SA-mediated resistance, were identified exclusively at 6 hpi. At 48 hpi, a Gas domain-containing protein (PVIT_0007104.T1), microtubule binding protein involved in growth and cell motility, and two Crinkler effectors (PVIT_0006424.T1 and PVIT_0001451.T1) were identified, being the first Crinkler effector also detected at 120 hpi.

### *Plasmopara viticola* proteins specifically found in the incompatible interaction with ‘Regent’

Two hundred and two proteins were exclusively found in the incompatible interaction between ‘Regent’ and *P. viticola*. Thirty-one proteins were predicted to be secreted and of those 17 were also predicted to be effector proteins. Hydrolases (excluding peptidases), transferases (including kinases) and oxidoreductases were identified at all the time-points (Supplementary Table S1).

**Figure 6:**
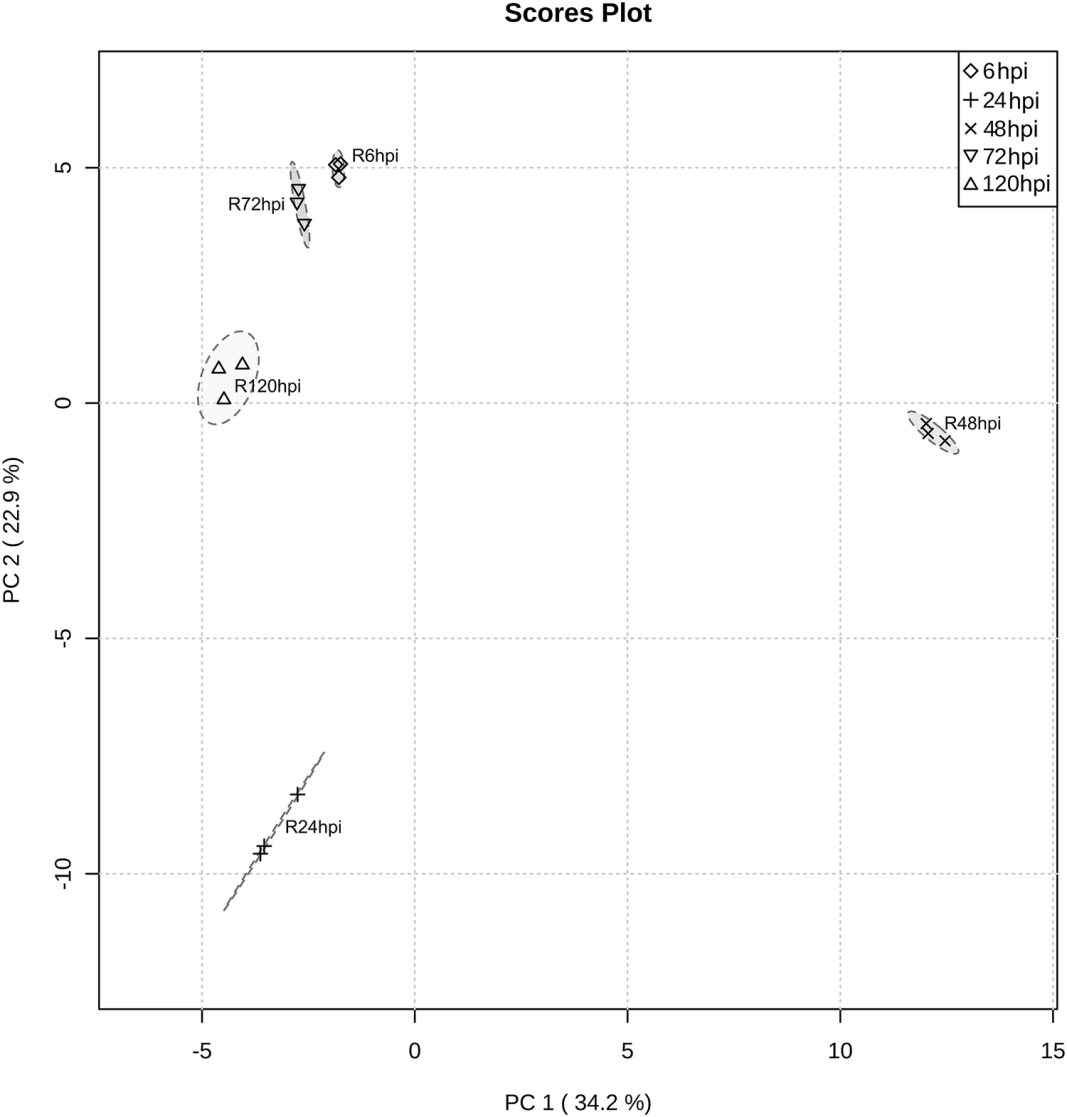
Principal components analysis (PCA) PC1/PC2 score plot of specific *P. viticola* proteome during interaction with *V. vinifera* cv. ‘Regent’. The three biological replicates per condition are represented.

When considering the proteins specific to this interaction, the unsupervised PCA bi-plot revealed that 24 and 48 hpi were the most distinct time-points, showing the highest levels of variation, while the other timepoints cluster together (Fig. 6). Twenty-three proteins contribute for the distinction of the 24 hpi proteome and 56 proteins for the 48 hpi proteome. At 24 hpi, a protein putatively involved in pathogen phosphoinositide pathway through phosphatidylinositol dephosphorylation (PVIT_0001582.T1) and a peptidase already described to be involved in bacteria virulence (PVIT_0007008.T1) were identified. At 48 hpi, an alternative oxidase (PVIT_0010523.T1), protein involved in pathogen defence against oxidative stress, a trehalose phosphatase (PVIT_0000913.T1), enzyme involved in the trehalose pathway, a phosphatidylserine decarboxylase (PVIT_0012236.T1), a developmental-related protein, and a Crinkler effector (PVIT_0006190.T1), were identified (Supplementary Table S1).

## Discussion

Plant pathogens cause important yield losses in crops. Thus, it is necessary to develop efficient and environmentally friendly crop protection strategies based on their biological cycles, virulence factors, and interaction with their hosts. The study of plant–pathogen interactions have been mainly focused on transcriptome analysis even though the transcript levels do not reflect protein levels and protein activity. Few studies describe the application of mass spectrometry-based proteomics to pathogens both in vitro and *in-planta*^19–21^. The average coverage of pathogen proteomes in an *in-planta* approach is of, approximately, 100 proteins^19^. In vitro proteomes enable a higher proteome coverage, from 283 to 1105^22^. This difference in proteome coverage may be explained by the limitations associated with the difference in biomass between host and pathogen present in the *in-planta* studies. To tackle this limitation, proteome may be sequenced from the host extracellular fluid. The extracellular fluid denotes the fluid present in the extracellular space (i.e. apoplast), the compartment where pathogens primary develops their infection structures. To the best of our knowledge, this approach was performed only once in *Cladosporium fulvum*-*Solanum lycopersicum* (tomato) interaction enabling the identification of 199 pathogen proteins^21^.

In the case of obligate biotrophic pathogens, as *P. viticola*, *in-planta* studies are the only solution as this pathogen only develops in living host tissues. After stomata penetration, the grapevine leaf extracellular space is the first compartment where *P. viticola* develops the primary infection structures until the establishment of complex hyphae for plant colonization^23,24^. Monitoring grapevine extracellular fluid proteins enables the *in-planta P. viticola* proteome characterization. Thus, in the present study, we have isolated the proteins present in the grapevine apoplastic fluid, from susceptible and *Rpv3*-mediated resistance cultivars, and identified a total of 420 *P. viticola* proteins.

### Characterization of *Plasmopara viticola* secretome and effectome

To further characterize the identified proteins, several tools for signal peptide, effector function and domain predictions were used. Seventy-nine *P. viticola* proteins presented signal peptide prediction for secretion, representing 19% of the total identified proteome. For other pathogens the percentage of proteins with signal peptide prediction for secretion varies from 30 to 60% of the total identified proteome. In *F. graminearum*, 56% of the 120 identified proteins *in-planta* were predicted to be secreted^25^. In *P. chlamydospore*, *P. gonapodyides* and *P. pseudosyringae*, between 56 and 60% of the identified proteins were predicted to be secreted^22^. In *P. infestans*, 316 proteins were predicted to be secreted, representing aproximately 30% of the 1105 proteins identified in vitro^26^. In this study, *P. viticola* showed a similar percentage of secreted proteins to *P. infestans* despite the different approaches used to identify their proteomes.

Regarding the identification of effectors, 164 proteins were predicted, which corresponded to 40% of the *P. viticola* total proteome. Moreover, of those, 48 of the identified effectors were predicted to be secreted (11% of the total). In *C. fulvum*-tomato interaction, 35% of total proteome, was considered as secreted protein effectors^21^. Effector proteins can be localized in either the apoplast or the cytoplasm of plant. Apoplastic effectors are secreted into the plant extracellular space, whereas cytoplasmic effectors are translocated into the plant cell, where they target different subcellular compartments^27–29^. Several apoplastic effectors contribute to counter-defence by inhibiting host enzymes, such as proteases and glucanases, that accumulate in response to pathogen infection^30^. Within the class of cytoplasmic effectors, the RXLR effectors, that contain the conserved RXLR-EER motif at N-terminal, have been reported to be widely distributed in oomycetes, including those from the genera *Phytophthora*, *Hyaloperonospora*, *Albugo*, and *Saprolegnia*. In *P. viticola*, only two proteins presenting the conserved RxLR-EER motif at the N-terminal were identified (PVIT_0014142.T1 and PVIT_0015177.T1). These proteins were only observed in the proteome of *P. viticola* during interaction with ‘Regent’. As a *Rpv3*-mediated resistance cultivar, ‘Regent’ can recognize the pathogen effectors through resistance proteins (R-proteins). This process results in an effector-triggered immunity (ETI) that ultimately leads to HR at the pathogen entry site^31^. Indeed, during infection of ‘Regent’ leaves with *P. viticola* necrotic spots are observed in the infection sites (Supplementary Fig. S1).

Crinkler proteins are another class of effectors widespread across the oomycete lineage, characterized by a LxLFLAK motif in the N-terminal, that can cause cell death^32^. In this study, four Crinkler proteins (PVIT_0001451.T1, PVIT_0006190.T1, PVIT_0006424.T1 and PVIT_0025443.T1) were identified in *P. viticola* proteome during both compatible and incompatible interactions. One of the identified Crinkler effectors, PVIT_0006424.T1, showed a slight difference in the conserved motif, where the phenylalanine (F) was substituted by a tyrosine (Y), resulting in a LxL**Y**LAK motif. This modification in the conserved motif of CRN effectors was already identified in another downy mildew pathogen, the *Plasmopara halstedii*^33^.

During the establishment of the *P. viticola* interaction with grapevine, hydrolases and transferases were the most observed enzymatic classes, independently of the type of interaction (compatible or incompatible). Within hydrolases, the class of peptidases were the most predominant subclass observed in *P. viticola*. Indeed, during the infection, plant pathogens secrete a large arsenal of hydrolytic enzymes, including peptidases. Peptidases are known to affect spore formation and germination and secreted peptidases may act as virulence factors. Additionally, peptidases from pathogens can inactivate or modify protein components of the host defence machinery and ultimately suppress defence responses. Several peptidase families were found to be a large part of oomycete secretomes, including aspartyl proteases, papain family cysteine proteases, subtilases, trypsins, and trypsin-like proteases^34^. In *P. viticola*, several serine and cysteine peptidases, including trypsin domain-containing proteins, calpain and papain family members, were identified. Within hydrolases, plant cell wall degrading enzymes, such as polygalacturonases, pectinesterases, pectin lyases, xylanases, and lipases, constitute another important group of secreted proteins that facilitates pathogen invasion. These enzymes have the ability to degrade plant cell walls, mainly composed by polysaccharides like cellulose, hemicelluloses, and pectin, facilitating the penetration and colonisation of host plants^35^. In *P. viticola* secretome, cellulase and pectinesterase domain-containing proteins were also identified. In *Magnaporthe oryzae*, cellulases contribute to the penetration of the host epidermis and to the further invasion of the phytopathogenic fungus^36^. Also, in *Botrytis cinerea*, the mutation of a gene encoding a pectinesterase led to a decrease in the virulence of this pathogen^37^.

Regarding the identification of transferases in *P. viticola* proteome during the infection process, around 50% of the proteins identified in this enzymatic class were kinases. Of those, several serine/threonine/tyrosine protein kinases presenting the PK_Tyr_Ser-Thr (PF07714) and the Pkinase (PF00069) domains, were identified. Protein kinases are a major class of signalling molecules that catalyse reversible phosphorylation of a large proportion of cellular proteins (∼30%), thereby modulating protein activity and gene expression. In pathogens, kinases pathways are involved in life cycle, stress responses and development, playing a pivotal role in the development of infection-related structures and virulence, as the well-known mitogen-activated protein kinase (MAPK) pathway. In *Saccharomyces cerevisiae*, MAPK cascades are essential for filamentation and invasive growth and regulate cell wall remodelling during the cell cycle and upon different stresses^38^. In *Colletotrichum* spp., reversible protein phosphorylation by kinases was described to be involved in the regulation of various growth and developmental processes associated with pathogenesis and responding to plant-surface signals^39^. Moreover, in oomycetes protein kinases have been shown to participate in the growth and differentiation of these pathogens. Indeed, in *P. infestans*, kinase inhibitors inhibited zoospore release, encystment, cyst germination, and reduced zoospore viability^40^.

### *Plasmopara viticola* phosphoinositide pathway seems to be important for the infection process

The analysis of *P. viticola* proteome revealed that several proteins related to the phosphoinositide pathway were accumulated differentially during the establishment of both interactions. At 6 hpi of ‘Trincadeira’ leaves, *P. viticola* accumulated a protein containing the Syja domain (PVIT_0014411.T1), which shows homology to the yeast protein SacI. This enzyme exhibits phosphatidylinositol polyphosphate phosphatase activity and can hydrolyse phosphate from any of the three positions of inositol that may be phosphorylated (3-, 4- and 5), originating phosphoinositide^41^. Phosphoinositides are key players in trafficking and signalling processes in eukaryotes that control membrane–cytoskeleton interactions and vesicle trafficking^42^. In *Colletotrichum higginsianum*, the enrichment of phosphoinositides has been shown to have a crucial role in pathogen infection^43^.

In *P. viticola* interaction with ‘Regent’, this phosphoinositide pathway seems to be closely related with clathrin-mediated endocytosis. This process is a vesicular transport event that facilitates the internalization and recycling of receptors engaged in a variety of processes, including signal transduction (G-protein and tyrosine kinase receptors), nutrient uptake and synaptic vesicle recycling^44^. The accumulation of phosphatidylinositol-4,5-bisphosphate (PIP2) and of adaptor proteins is required for the formation of clathrin complex. Then, a reorganization of the cytoskeleton is required for growth and transport of the clathrin-coated vesicles. Based on the receptor type or on the membrane composition, the vesicles are transported to the various destinations including the trans-Golgi network, endosomes, and vacuoles^44^. During infection of ‘Regent’ leaves, these clathrin adaptor proteins were identified until 48 hpi (PVIT_0013762.T1 at 6 hpi and PVIT_0006099.T1 at 24 and 48 hpi). At 24, 72 and 120 hpi, a phosphatidylinositol-4-phosphate 5 kinase (PVIT_0002180.T1), a protein that catalyses the synthesis of PIP2 was identified. Moreover, at 24 and 120 hpi, a calmodulin-regulated spectrin-associated protein (CAMSAP; PVIT_0012402.T1), protein related with cytoskeleton dynamics which acts on microtubule ends to regulate their dynamics, was identified^45^. Furthermore, at 120 hpi, a tubulin (PVIT_0002309.T1) and a kinesin (PVIT_0011113.T1) domain containing proteins were identified in *P. viticola* proteome during infection of ‘Regent’ leaves. Tubulins are major components of the microtubules that are involved in many cellular processes, such as cell division, ciliar or flagellar motility and intracellular transport in eukaryotic organisms^46^. Kinesins are microtubule-associated force-producing proteins that may play a role in organelle transport. In fungi, kinesins have been reported to play an important role in morphogenesis of fungal hyphae^47^. However, in oomycetes, the role of these proteins is not yet elucidated.

Phosphoinositide pathway is activated differently according to the interaction thus reflecting different strategies to improving signalling and vesicular trafficking for development of the infection.

### *Plasmopara viticola* may interfere with host salicylic-acid pathway and use host isoprenoids to establish a compatible interaction

Host hormonal pathways and isoprenoid associated compounds have been shown to be modulated as a defence strategy after *P. viticola* infection^48,49^. Nonetheless, recent evidence points out that oomycete’s effectors are able to modulate host phytohormone pathways by directly altering hormone levels or interfering with hormonal biosynthetic and signalling pathways^50^. Within *P. viticola* proteome, at 6 h of interaction with the susceptible cultivar ‘Trincadeira’ an ENHANCED DISEASE RESISTANCE 2 (EDR2) domain containing protein (PVIT_0008910.T1) was identified. This protein has been described as a negative regulator of salicylic acid (SA)-mediated resistance limiting cell death and HR establishment in plant cells^51^. Also, a NUDIX domain containing protein (PVIT_0025277.T1) was identified. These proteins were shown to modulate SA signalling pathway through negative regulation of the ENHANCED DISEASE SUSCEPTIBILITY1 (EDS1) signalling pathway^52^. Moreover, at 24 and 48 hpi, a diacylglycerol kinase (PVIT_0020186.T1) was identified. This enzyme catalyses the phosphorylation of diacylglycerol to phosphatidic acid, thus modulating the balance between the two signalling lipids^53^. Phosphatidic acid is one of the possible ligands of EDR2 via both its PH and START domains. EDR2 was shown to influence plant SA pathway through direct or indirect inhibitory effect on EDS1 via a lipid-like intermediate, like sphingolipid, phosphatidic or oleic acids. The identification of negative regulators of EDS1 and SA pathways reinforces the hypothesis that *P. viticola* may overcome grapevine defences by decreasing the levels of host SA.

Oomycetes are sterol auxotrophs i.e., they are unable to synthesize sterol molecules, but they can use host sterols to support mycelium development and reproduction^54,55^ and also to produce pathogenesis related compounds, namely elicitins through the redirection of host mevalonate pathway flux^56^. Elicitins are extracellular proteins already shown to be produced by *Phytophthora* sp. and other *Peronosporales* genera^57^ that act as sterol carriers and are important in infection development^58^. Elicitin-mediated acquisition of plant sterols is required for growth and sporulation of *Phytophthora* spp.^59^. In the compatible grapevine-*P. viticola* interaction, two pathogen proteins related with the synthesis of isoprenoid sterols were found: at 6 hpi a polyprenyl synthetase (PVIT_0015689.T1) and at 72 hpi a mevalonate diphosphate decarboxylase (PVIT_0016187.T1). Thus, these results may suggest that *P. viticola* is re-directing grapevine mevalonate pathway to synthesize isoprenoid sterols, essential for its growth and development.

### Specific development-related proteins are more prominent in *Plasmopara viticola* proteome during the incompatible interaction

During *P. viticola* infection of the *Rpv3*-mediated resistance cultivar ‘Regent’, several proteins related to pathogen development, were exclusively found in this incompatible interaction. As soon as 6 hpi and later as 120 hpi, a Gaa1 domain-containing protein (PVIT_0009119.T1) was detected. Gaa1 together with Gpi8, Gpi16, Gab1, and Gpi17 form the glycosylphosphatidylinositol (GPI) transamidase complex^60^. This enzyme catalyses the attachment of GPI units to the C-terminal of newly synthesised proteins. In *Colletotrichum graminicola*, Gaa1 is required for differentiation of functional appressoria, vegetative hyphal growth and asexual sporulation, having an important role in this pathogen infection mechanism^61^. In *P. viticola*, it is possible to hypothesize that Gaa1 might be involved in appressoria and hyphal growth in the first hours of infection and, at a later stage of the infection, in the asexual sporulation of the pathogen for subsequent reinfection. At 24 and 48 hpi, two developmental-related proteins were identified, a phosphatidate phosphatase APP1 (PVIT_0002769.T1) and a phosphatidylserine decarboxylase (PVIT_0012236.T1). In *Candida albicans*, a phosphatidate phosphatase plays an important role in hyphal growth, adaptability to environmental stresses, and virulence^62^, and a phosphatidylserine decarboxylase is also required for virulence^63^. At later time-points (72 and 120 hpi), an annexin (PVIT_0006991.T1) was identified in *P. viticola* proteome during the incompatible interaction. This protein function as (1 → 3)-β-d-glucan synthase activator in oomycetes, thus it is potentially involved in the regulation and development of the cell wall of these organisms during the infection process^64^.

### Glucose and trehalose pathways are actively modulated in *Plasmopara viticola* during the incompatible interaction

In the incompatible interaction between *P. viticola* and ‘Regent’, a glucose-6-phosphate dehydrogenase (G6PDH; PVIT_0011126.T1) was identified as soon as 6 hpi. G6PDH is the key enzyme supplying reducing power (NADPH) to the cells. In turn, NADPH/NADP ratio is involved in regulating the expression of virulence-associated genes which play a role in appressorium formation^65^. At 48 hpi, a trehalose phosphatase (PVIT_0000913.T1), enzyme responsible for the phosphorylation of trehalose-6-phosphate to trehalose, was identified. Trehalose is an important signalling metabolite that might be involved in the regulation of carbon metabolism and photosynthesis^66^. Also, it seems to play an important role in plant-pathogen interactions. Indeed, plant pathogens can accumulate higher amounts of trehalose than can normally be found in plants^65^. In Arabidopsis, *Plasmodiophora brassicae* mediates trehalose accumulation in plant infected organs through the upregulation of its own trehalose-6-phosphate synthase gene. From the host side, trehalase gene becomes up-regulated as a defence mechanism to prevent excess accumulation of trehalose in the infected cells^67^. In the rice blast fungus *Magnaporthe grisea*, the trehalose biosynthetic pathway was shown to be required for colonization of the plant tissue^68^. Moreover, in susceptible *Citrus* spp, the expression of genes encoding trehalose-6-phosphate synthase and trehalose phosphatase was highly induced during bacterial infection with *Xanthomonas citri* subsp. *citri*. Also, in this interaction, results suggested that bacterial driven trehalose plays a role in modifying the host plant’s metabolism to its own advantage^69^. Thus, pathogen trehalose seems to be associated to plant metabolism modulation in a way that favoured pathogen development. In contrast, exogenous trehalose can improve resistance of plants, reducing stomatal aperture, up-regulate NADPH oxigenase genes and increase ROS^70^. So, it is possible to hypothesize that a tight regulation of this signalling molecule in both plant and pathogen cells is important. In *P. viticola*, the G6PDH is also identified in later time-points as 72 and 120 hpi. A cooperative regulation of both glucose and trehalose pathways must be happening to help the infection mechanism of *P. viticola* while trying not to activate the grapevine defences.

### *Plasmopara viticola* enhances the accumulation of oxidative stress defence-related proteins during infection of ‘Regent’ leaves

During the establishment of an incompatible interaction, one of the most common mechanisms of plant defence is the production and accumulation of ROS near the infection sites^71–73^. In that way, plant pathogens must protect themselves from the oxidative stress generated by plant ROS. In *P. viticola*, a catalase (PVIT_0009071.T1) was identified at 24 and 48 hpi on ‘Regent’ leaves. This enzyme acts as an antioxidant, and it is involved in the conversion of hydrogen peroxide to water and oxygen protecting cells from its toxic effects. In *Phytophthora nicotianae*, an in vitro assay showed that the activity of a catalase peaks in sporulating hyphae. Also, in an *in-planta* assay with tomato leaves the catalase activity increased dramatically about 8 h after host inoculation^74^. This behaviour is consistent with the role of pathogen catalase in counter defence and protection against oxidative stress. At 48 hpi, an alternative oxidase (PVIT_0010523.T1), a ubiquinol terminal oxidase that is involved in mitochondrial oxidative phosphorylation, was identified. In *Cryptococcus neoformans*, an alternative oxidase has a role in the yeast's defence against exogenous oxidative stress and contributes to the virulence of this organism^75^. In *B. cinerea*, mutants for this enzyme exhibited defects in mycelial growth, sporulation, spore germination, and virulence, highlighting its role and importance in pathogen infection mechanism^76^. In *P. viticola*, the accumulation of these proteins suggests an effort from this oomycete to protect from the oxidative stress caused by grapevine, as a plant defence mechanism, during the development of *P. viticola* infection structures.

## Conclusions

Uncovering pathogen infection mechanisms in plants with different degrees of susceptibility and tolerance could help the identification of key-molecules or pathways that can be targeted for the development of new sustainable plant disease control strategies. Our results showed that *P. viticola* behaves differently depending on the interaction established with the grapevine genotypes, and this is reflected at the proteome level, namely in the secretome and effectome profiles. Moreover, *P. viticola* seems to activate its own phosphoinositide pathway in different ways, depending on the type of interaction being established, to improve signalling and vesicular trafficking for development and growth. During the compatible interaction, *P. viticola* can interfere with salicylic acid pathway and interfere with isoprenoid biosynthesis of the plant, suggesting an effort of this pathogen to repress the plant defences to successfully colonize it. In contrast, in the incompatible interaction, the proteins found suggested that *P. viticola* is encountering more challenges to differentiate the infection structures, while trying to manipulate host metabolism to its favour and protecting itself from the oxidative stress generated by plant defences responses.

## Materials and methods

### Plant material

As model of a compatible interaction between grapevine and *P. viticola*, the *V. vinifera* cv ‘Trincadeira’, a Portuguese elite cultivar highly susceptible to *P. viticola*, was selected. For the analysis of an incompatible interaction, the *V. vinifera* cv ‘Regent’ (VIVC number 4572), a Germany crossing line, was used. ‘Regent’ results from the cross between *V. vinifera ssp vinifera* cv. ‘Diana’ and the interspecific hybrid ‘Chambourcin’ (created by Professor Gerhardt Alleweldt, in 1967, at the Geilweilerhof Institute for Grape Breeding, Germany). ‘Regent’ is one of the most cultivated downy mildew resistant varieties in Europe^77,78^, and carries the dominant Rpv3-1 loci, conferring tolerance to *P. viticola* infection.

Hundred and fifteen wood cuttings from both cultivars were obtained from the commercial nursery VitiOeste (Pó-Bombarral, Portugal) and grown in 2.5 L pots in universal substrate under controlled conditions in a climate chamber at natural day/night rhythm, relative humidity 60% and a photosynthetic photon flux density of 300 μmol m^−2^ s^−1^.

All methods were carried out in accordance with relevant institutional, national, and international guidelines and legislation guidelines complying with the Convention on Biological Diversity (https://www.cbd.int/convention/) and the Convention on the Trade in Endangered Species of Wild Fauna and Flora (https://cites.org/eng).

### Plant inoculation with *Plasmopara viticola*

For plant inoculation, downy mildew symptomatic leaves from susceptible plants were harvested in the field at the Portuguese Grapevine Germplasm Bank (PRT051), sprayed with water, and incubated overnight at 22 °C in the dark to enhance sporulation. Then, next day, *P. viticola* sporangia were collected and their vitality was checked by microscopy, as described in^79^. Inoculum was propagated in the laboratory using the susceptible *V. vinifera* cv Müller-Thurgau by spreading a sporangia solution on the abaxial surface of the leaf, with an undefined concentration of *P. viticola* sporangia. After infection, leaves were kept in the dark for the first 8 to 12 h and then incubated at 25 °C with natural light conditions, until sporulation was observed. Spores were collected with a vacuum system and stored at − 20 °C until further use.

For the experimental assay, ‘Regent’ and ‘Trincadeira’ abaxial leaf surfaces were sprayed with an inoculum solution containing 3.5 × 10^5^ sporangia mL^−1^. Mock-inoculations with water were also made and used as control. After inoculation, plants were kept in a greenhouse under high humidity conditions and 25 °C. The third to fifth fully expanded leaves beneath the shoot apex were harvested at 6, 24, 48, 72 and 120 h post inoculation for apoplast fluid extraction. Leaf material was also collected and immediately frozen in liquid nitrogen and kept at − 80 °C for RNA extraction.

### *Plasmopara viticola* RNA Extraction and cDNA Synthesis

Total RNA was isolated from 100 mg of frozen leaves with the Spectrum™ Plant Total RNA Kit (Sigma-Aldrich, USA), according to manufacturer's instructions. Residual genomic DNA was digested with DNase (TURBO DNA-free™ Kit, Thermo Scientific, USA). RNA purity and concentration were measured at 260/280 nm using a spectrophotometer (NanoDrop-1000, Thermo Scientific) while RNA integrity was verified by agarose gel electrophoresis (1.2% agarose in TAE buffer). Genomic DNA (gDNA) contamination was checked by qPCR analysis of a target on the crude RNA^80^. Complementary DNA (cDNA) was synthesized from 2.5 µg of total RNA using RevertAid®H Minus Reverse Transcriptase (Fermentas, Ontario, Canada) anchored with Oligo(dT)23 primer (Fermentas, Ontario, Canada), according to manufacturer's instructions. Three biological replicates were considered.

### Quantitative Real Time PCR analysis

Quantitative real time-PCR (qPCR) experiments were carried out using Maxima™ SYBR Green qPCR Master Mix (2×) kit (Fermentas, Ontario, Canada) in a StepOne™ Real-Time PCR system (Applied Biosystems, Sourceforge, USA). A final concentration of 2.5 mM MgCl_2_ and 0.2 μM of each primer were used in 10 μL volume reactions, together with 1 μL of cDNA (diluted 1/10) as template. A control without cDNA template was included in each set of reactions. Primer sequences and reaction details are provided in Supplementary Table S5.

Thermal cycling for all genes started with a denaturation step at 95 °C for 10 min followed by 40 cycles of denaturation at 95 °C for 15 s and annealing at 60 °C for 30 s. Dissociation curves were used to analyse non-specific PCR products. Three biological replicates and two technical replicates were used for each sample. Expression profiles for *PvRxLR28* (KX010958.1) and *PvRxLR67* (KX010967.1) were calculated using ΔCt method and *Pvactin* (HE582092.1) as reference gene.

### Extraction of grapevine leaf apoplastic fluid

Apoplastic fluid extraction of ‘Regent’ and ‘Trincadeira’ leaves (inoculated and mock-inoculated samples) was performed as described in^81^. Briefly, for each condition, 30 g of leaves were vacuum infiltrated with 0.1 M Tris–HCl buffer (pH 8.0) solution containing 0.5 M KCl, 0.006 M CHAPS and 2% (w/v) Na_2_SO_3_ (at 4 °C), for six cycles of 30 s, and the apoplastic fluid recovered by centrifugation (5000 g, 15 min at 4 °C). Three biological replicates were considered.

### *Plasmopara viticola* protein identification by mass spectrometry

Protein separation by liquid chromatography and their mass spectrometry-based identification were performed as described in^81^. Briefly, twenty micrograms of proteins were digested then extracted peptides were separated and identified by nanoLC-MS/MS. For identification of *P. viticola* proteins, the genome assembly of *Plasmopara viticola* genome database (INRA-PV221 isolate; 15 960 sequences, April 2018, https://doi.org/10.15454/4NYHD6) was used via Mascot Daemon interface (v.2.6.0. Matrix Science), the identification results were imported to Progenesis QIP and matched to peptide spectra. Only proteins with a minimum of two matching sequences and one unique sequence per identified protein, and with a significance MASCOT-calculated threshold *p*-value < 0.05, were considered.

Functional information about *P. viticola* proteins was obtained from *P. viticola* genome database. Protein secretion was predicted using SignalP 5.0, TargetP 2.0 (http://www.cbs.dtu.dk/services/,^82,83^) and Phobius (http://phobius.sbc.su.se/,^84^). For effector function prediction EffectorP 3.0 (http://effectorp.csiro.au/,^85^) and EffHunter Pipeline^86^ were used. EffectorP is a machine learning method for fungal and oomycete effector prediction in secretomes that recognizes cytoplasmic and apoplastic signals, including in non-secreted intracellular proteins^85^. EffectorP expands predicted effector repertoires beyond small, cysteine-rich secreted proteins in fungi and RxLR-motif containing secreted proteins in oomycetes. EffHunter Pipeline is based on four criteria for definition of an effector protein: (1) protein length ≤ 400 amino acids; (2) presence of signal peptide; (3) absent of transmembrane domains; (4) presence of ≥ 4 cysteine residues^86^. Only proteins that fulfil these criteria at the same time are predicted to be effectors. Phobius was also used for transmembrane domain prediction. Cysteine content was calculated through EMBOSS Pepstats tool (https://www.ebi.ac.uk/Tools/seqstats/emboss_pepstats/,^87^). For domain prediction, hidden Markov Models tool from Pfam database (https://www.ebi.ac.uk/Tools/hmmer/search/hmmscan,^88^) was used. For effector motifs prediction, the R package *effectR* was used. The *effectR* package relies on a combination of regular expressions statements and hidden Markov model approaches to predict candidate RxLR and Crinkler effectors^16^.

For representation purposes, calculations were done to present the number of secreted proteins, effector proteins and enzymatic classes as follows.

For each grapevine cultivar and time-point of infection, the number of proteins predicted to be secreted was divided by the total number of proteins identified, resulting in the percentage of secreted proteins for each experimental condition (represented in Fig. 1), as following:

$$\% secreted\;proteins\;per\;experimental\;condition = \frac{{\mathop \sum \nolimits_{n = 3} secreted\;proteins}}{{\mathop \sum \nolimits_{n = 3} total\;identified\;proteins }} \times 100$$

For each grapevine cultivar and time-point of infection, the number of proteins predicted to be effector proteins was divided by the total number of proteins identified, resulting in the percentage of effector proteins for each experimental condition (represented in Fig. 2), as following:

$$\% effector\;proteins\;per\;experimental\;condition = \frac{{\mathop \sum \nolimits_{n = 3} effector\;proteins}}{{\mathop \sum \nolimits_{n = 3} total\;identified\;proteins }} \times 100$$

For each grapevine cultivar, the number of proteins belonging to each of the seven enzymatic classes was divided by the total number of proteins identified, resulting in the percentage of proteins of each enzymatic class for each cultivar, independently of the time-point of infection where they were identified (represented in Fig. 3), as following:

$$\% enzymes\;of\;each\;class\;per\;cultivar = \frac{{\mathop \sum \nolimits_{n = 3} enzymes\;of\;each\;class}}{{\mathop \sum \nolimits_{n = 3} total\;identified\;proteins }} \times 100$$

For each grapevine cultivar and time-point of infection, the number of proteins belonging to each of the seven enzymatic classes was divided by the total number of proteins identified, resulting in the percentage of proteins of each enzymatic class for each experimental condition (represented in Fig. 4), as following:

$$\% enzymes\;of\;each\;class\;per\;experimental\;condition = \frac{{\mathop \sum \nolimits_{n = 3} enzymes\;of\;each\;class}}{{\mathop \sum \nolimits_{n = 3} total\;identified\;proteins }} \times 100$$

### Statistical analysis

Principal Component Analysis (PCA) of *P. viticola* proteomes during interaction with ‘Trincadeira’ and ‘Regent’ leaves was performed using the program MetaboAnalyst 5.0 (http://www.metaboanalyst.ca/,^89^).

## Supplementary Information

Supplementary Information 1.

Supplementary Information 2.

Supplementary Information 3.

Supplementary Information 4.

Supplementary Information 5.

Supplementary Information 6.

## Data Availability

The mass spectrometry proteomics data is deposited in the ProteomeXchange Consortium via the PRIDE partner repository with the dataset identifier PXD030508 and https://doi.org/10.6019/PXD030508 (https://www.ebi.ac.uk/pride/archive/projects/PXD030508).
