# Supplementary material for: An in-planta comparative study of Plasmopara viticola proteome reveals different infection strategies towards susceptible and Rpv3-mediated resistance hosts
Source: Sci Rep. 2022 Dec 1;12:20794. doi: 10.1038/s41598-022-25164-8 (PMC9715676; doi:10.1038/s41598-022-25164-8)
Supplement: Supplementary file 1 — Supplementary Information 1. [file 41598_2022_25164_MOESM1_ESM.pdf]

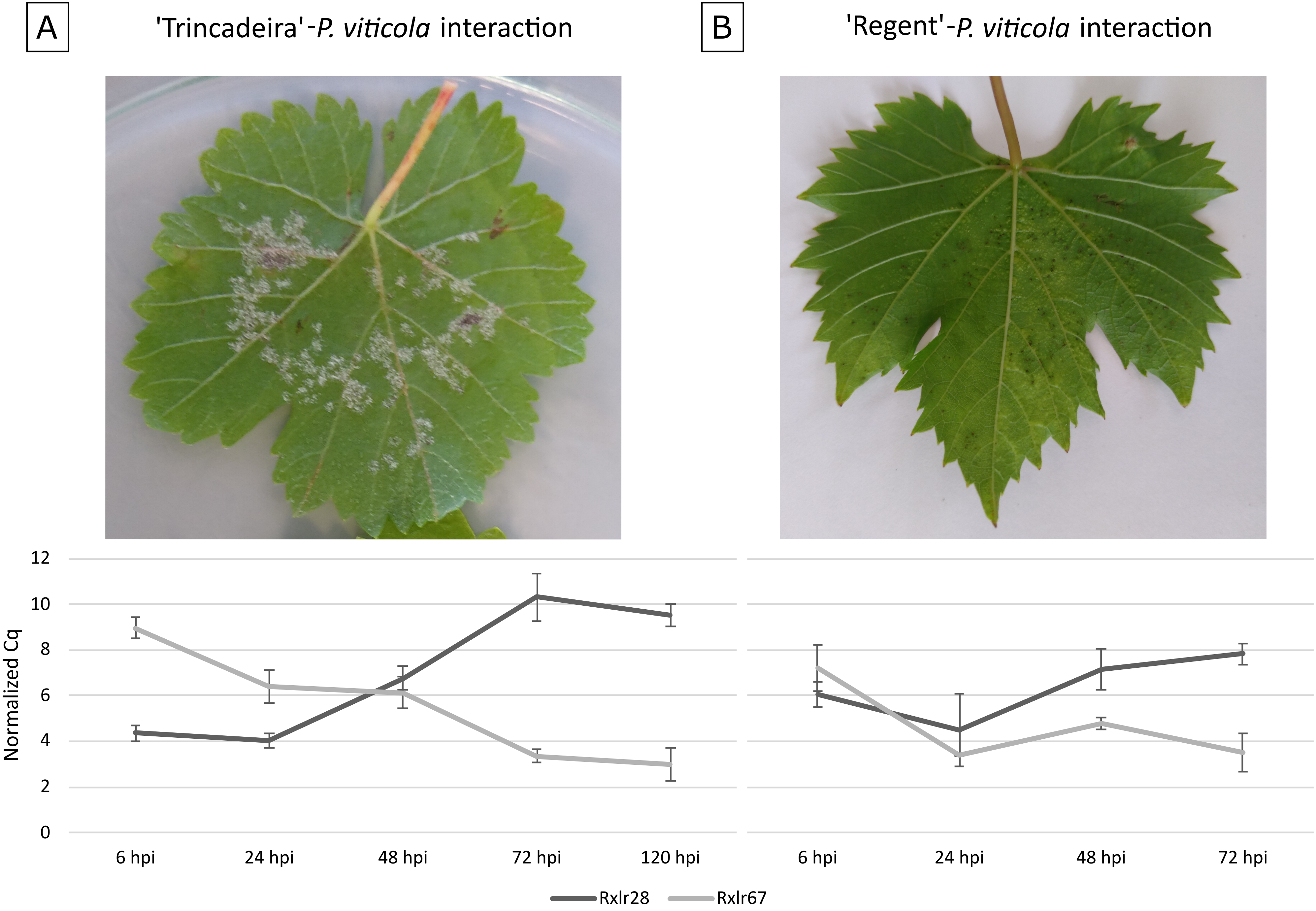

Supplementary Figure S1. Representative images of leaf symptoms caused by downy mildew disease and normalized quantification cycle (Cq) values for *P. viticola* effectors *PvRxLR28* (dark grey line) and *PvRxLR67* (light grey line) in 'Trincadeira'-*P. viticola* (A) and 'Regent'-*P. viticola* (B) interactions. No gene expression was detected in mock-inoculated controls. A low Cq corresponds to a high amount of target mRNA whereas a higher Cq corresponds to a lower amount of target mRNA.
