# Supplementary material for: An in-planta comparative study of Plasmopara viticola proteome reveals different infection strategies towards susceptible and Rpv3-mediated resistance hosts
Source: Sci Rep. 2022 Dec 1;12:20794. doi: 10.1038/s41598-022-25164-8 (PMC9715676; doi:10.1038/s41598-022-25164-8)
Supplement: Supplementary file 3 — Supplementary Information 3. [file 41598_2022_25164_MOESM3_ESM.pdf]

Supplementary Table S2. *Plasmopara viticola* proteins belonging to group 1 of proteins identified in both interactions at 48 hpi. Protein accession (*Plasmopara viticola* genome database), enzymatic classification (based on protein domains), signal peptide, effector and domain predictions and abundance value are represented.

Accession	Enzyme classification	Signal peptide prediction	Effector prediction	Domains	Abundances	
					'Trincadeira'	'Regent'
PVIT_0002749.T1	Ligase	No	No	ACC_central (PF08326); Carboxyl_trans (PF01039); CPSase_L_D2 (PF02786); Biotin_carb_N (PF00289); Biotin_carb_C (PF02785); Biotin_lipoyl (PF00364)	38,8	21,0
PVIT_0010364.T1	Oxidoreductase	No	Yes	adh_short (PF00106); adh_short_C2 (PF13561)	175,7	1102,3
PVIT_0007744.T1	Other	No	No	STAG (PF08514)	284,7	1032,3
PVIT_0001847.T1	Cell development	No	No	WD40 (PF00400); FOP_dimer (PF09398); LisH_2 (PF16045); Nup160 (PF11715)	25,1	613,6
PVIT_0000553.T1	Transferase	Yes	No	HECT (PF00632)	2,9	4,3
