# Supplementary material for: An in-planta comparative study of Plasmopara viticola proteome reveals different infection strategies towards susceptible and Rpv3-mediated resistance hosts
Source: Sci Rep. 2022 Dec 1;12:20794. doi: 10.1038/s41598-022-25164-8 (PMC9715676; doi:10.1038/s41598-022-25164-8)
Supplement: Supplementary file 4 — Supplementary Information 4. [file 41598_2022_25164_MOESM4_ESM.pdf]

Supplementary Table S3. *Plasmopara viticola* proteins belonging to group 2: proteins observed at early time-points in the compatible interaction and detected at later time points in the incompatible interaction. Protein accession (*Plasmopara viticola* genome database), enzymatic classification (based on protein domains), signal peptide, effector and domain predictions and abundance value are represented.

**Accession**	**Enzyme classification**	**Signal peptide prediction**	**Effector prediction**	**Domains**	**'Trincadeira'**	**'Regent'**			
					**6h**	**24h**	**48h**	**72h**	**120h**
PVIT_0006618.T1	Ligase	No	No	tRNA-synt_1 (PF00133); tRNA-synt_1g (PF09334); tRNA-synt_1e (PF01406); Anticodon_1 (PF08264)	323,6		117,5		
PVIT_0001990.T1	Kinase	No	Yes	Pkinase (PF00069); PK_Tyr_Ser-Thr (PF07714); Pkinase_fungal (PF17667)	45,6		5,0		
PVIT_0005502.T1	Other	Yes	Yes	Pho88 (PF10032)	47,6		2,0		
PVIT_0005313.T1	Other	Yes	No	Na_H_Exchanger (PF00999)	39,9		174,0		
PVIT_0008845.T1	Other	No	No	Ank (PF00023); Ank_2 (PF12796); Ank_3 (PF13606); Ank_4 (PF13637); Ank_5 (PF13857)	18,1		142,6		
PVIT_0004400.T1	Transferase	No	No	DNA_pol_phi (PF04931)	214,2			1,1	
PVIT_0011238.T1	Hydrolase	Yes	No	GTP_EFTU (PF00009); GTP_EFTU_D2 (PF03144); GTP_EFTU_D3 (PF03143)	1254,4				6,6
