# Supplementary material for: An in-planta comparative study of Plasmopara viticola proteome reveals different infection strategies towards susceptible and Rpv3-mediated resistance hosts
Source: Sci Rep. 2022 Dec 1;12:20794. doi: 10.1038/s41598-022-25164-8 (PMC9715676; doi:10.1038/s41598-022-25164-8)
Supplement: Supplementary file 5 — Supplementary Information 5. [file 41598_2022_25164_MOESM5_ESM.pdf]

Supplementary Table S4. *Plasmopara viticola* proteins belonging to group 2: proteins observed in early time-points in the incompatible interaction and detected at later time-points in the compatible interaction. Protein accession (*Plasmopara viticola* genome database), enzymatic classification (based on protein domains), signal peptide, effector and domain predictions and abundance value are represented.

**Accession**	**Enzyme classification**	**Signal peptide prediction**	**Effector prediction**	**Domains**	**'Regent'**	**'Trincadeira'**		
					**6h**	**24h**	**48h**	**120h**
PVIT_0013015.T1	Kinase	No	No	Pkinase (PF00069); PK_Tyr_Ser-Thr (PF07714); RyR (PF02026)	5,5	131,8		
PVIT_0001556.T1	Hydrolase	No	Yes	UCH (PF00443)	12,0		440,4	
					**24h**			
PVIT_0003475.T1	Other	No	No	Myotub-related (PF06602); FYVE (PF01363); C2 (PF00168)	4,9		29,2	
PVIT_0005567.T1	Hydrolase	No	Yes	Gtr1_RagA (PF04670); Arf (PF00025); Roc (PF08477); Ras (PF00071); MMR_HSR1 (PF01926)	38,3		44,4	
					**48h**			
PVIT_0017117.T1	Other	Yes	No	SKN1_KRE6_Sbg1 (PF03935)	562,9			157,9
					**72h**			
PVIT_0007479.T1	Other	Yes	Yes	Mito_carr (PF00153)	9,8			344,9
