# Supplementary material for: An in-planta comparative study of Plasmopara viticola proteome reveals different infection strategies towards susceptible and Rpv3-mediated resistance hosts
Source: Sci Rep. 2022 Dec 1;12:20794. doi: 10.1038/s41598-022-25164-8 (PMC9715676; doi:10.1038/s41598-022-25164-8)
Supplement: Supplementary file 6 — Supplementary Information 6. [file 41598_2022_25164_MOESM6_ESM.pdf]

Supplementary Table S5. Genes, primers and amplification information for the qPCR analysis.

Gene Name<br>NCBI Accession	Primer sequence 5’-3’	Amplicon (bp)	Amplification efficiency (%)	Ta (°C)	Tm (°C)
Actin<br>HE582092.1	F: CTCACGTACATTGCCTTGGA<br>R: AATACCTGACGCTTCTTTACC	177	104	60	80.49
PvRxLR28<br>KX010958.1	F: AACGTGGACGAAGATAAGGGA<br>R: AATTTTCAAAGGGTGGGATAC	109	136	60	78.70
PvRxLR67<br>KX010967.1	F: TGCACCAAGAATCCAAGAAGT<br>R: ATGCGGCGCTCAAACAATG	90	99	60	76.92

Ta, annealing temperature; Tm, melting temperature.
